# Intrathoracic Defects—A Reconstructive Approach

**Published:** 2014-07-26

**Authors:** James Allan, Jeon Cha, Frank Hsieh, Johnny Kwei, John G. Vandervord

**Affiliations:** Burns, Plastic, Reconstructive and Maxillofacial Surgery, Royal North Shore Hospital, St Leonards, Sydney, Australia

**Keywords:** intrathoracic defect, bronchopleural fistula, empyema, pectoralis major, flap reconstruction

## DESCRIPTION

**Figure 1:**
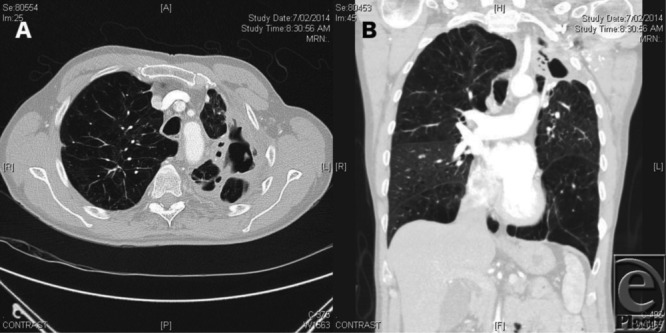
(*a*) Axial slice of contrast enhanced computer tomography of the upper lobe. (*b*) Coronal slice of a contrast enhanced computer tomography of the chest. Both slices demonstrate diseased lung tissue in the left upper lobe and an infective collection in the pleural space.

A 61-year-old man with a cavitating Mycobacterium Kansasii lesion and chronic hemoptysis underwent bronchial artery embolization and subsequently developed an infective collection in the pleural space and a persistent diseased left upper lobe (Fig 1). This longstanding condition was eventually treated with a left upper lobectomy.

## QUESTIONS

1. **What surgical options are available to deal with a pleural defect?**
2. **At what sites are locoregional flaps delivered into the thoracic cavity?**
3. **What are the surface markings for the major pedicle of pectoralis major?**
4. **How do you denervate pectoralis major?**

## DISCUSSION

**Figure 2:**
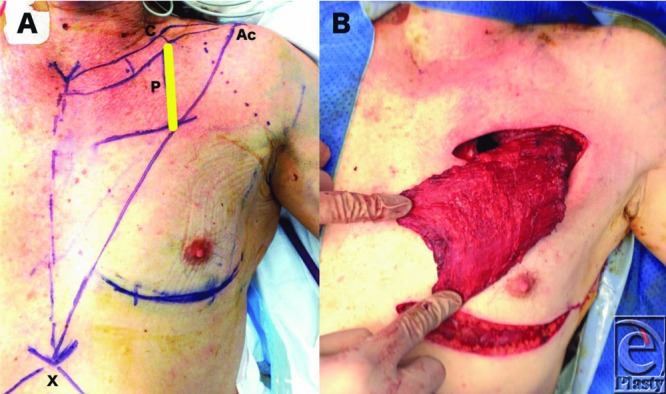
(*a*) The line P demonstrates the surface marking of the thoracoacromial trunk (C = junction of middle and lateral third of the clavicle, Ac = acromion, X = xiphisternum. (*b*) Pectoralis major elevated off its attachments with exposure through two transverse incisions.

Intrathoracic defects can be the result of trauma or surgical resections. Lung resections are performed most frequently for clearance of abnormal lung tissue (eg, emphysema), benign tumors (eg, carcinoid), and lung cancer and infections (eg, tuberculosis, abscesses, fungi). The potential adverse sequelae from either lobectomies and pneumonectomies are bronchopleural fistulas (occurring in 1.5%-28%) and empyemas (occurring in 1%-11%).^1^ When an empyema does occur, it may pose a significant risk to the patient with mortality rates between 16% and 40% being reported.^2^^,^^3^ Consequently, the potential space resulting from these procedures needs to be considered and addressed. The Clagett principle for treating pleural defects involves formalizing a pleural-cutaneous fistula with a large window and allowing free drainage until healthy granulation tissue is formed. The wound is then closed without obliteration of dead space.^4^ In 1911, Abrashanoff^5^ described using local muscle flaps to close a bronchopleural fistula. With better understanding of the vascularity of tissues and with the advent of microsurgery techniques, our options for filling these defects are now composed of locoregional and distant flaps. Locoregional flap options include serratus anterior, latissimus dorsi, pectoralis major (Fig 2), intercostals, rectus abdominus, and omentum.^1^^-^3 Distant reconstructive options may be required due to a deficiency of locoregional options resulting from trauma, multiple thoracotomies, and inadequate bulk. The recipient vessels of choice are the thoracodorsal artery and thoracoacromial trunk as they are located outside the thoracic cage and can be covered with muscle to protect the anastomosis.^1^

**Figure 3:**
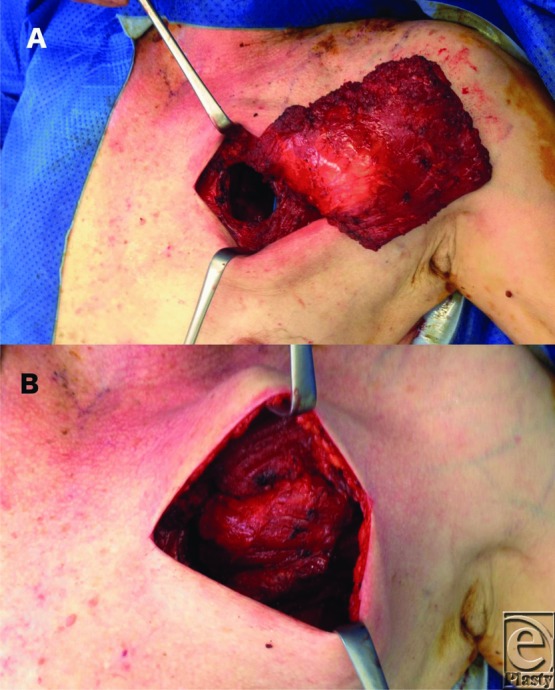
(*a*) Anterior aspect of pleural cavity has been approached following the resection of a segment of the 3rd rib. (*b*) Pectoralis major is delivered into the pleural space via the anterior window.

**Figure 4:**
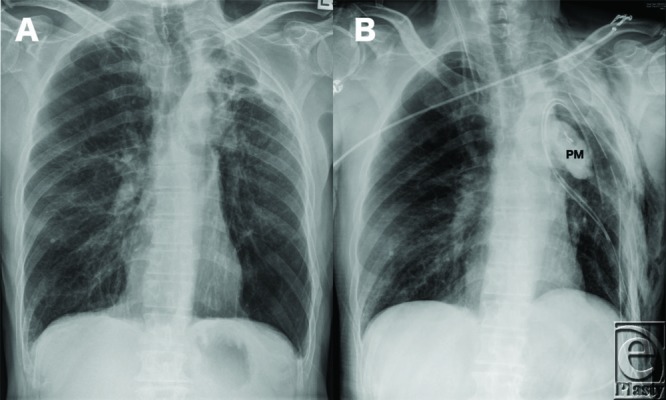
(*a, b*) Preoperative and postoperative plain chest x-rays. The pectoralis major (PM) flap obliterating the intra-thoracic dead space resulting from the upper lobectomy.

In 1938, Gray^6^ described a small thoracotomy window for transposing a latissimus dorsi into a pleural defect. The thorax can be entered at numerous sites including using the same access routes as the cardiothoracic team if appropriate (posterolateral thoracotomy incisions—sparing the latissimus dorsi and the serratus anterior or through a median sternotomy incision).^3^ When this is not suitable, serratus anterior and latissimus dorsi flaps are usually delivered to the intrathoracic defect via an opening formed by resection of 1 or 2 ribs at the level of the previous thoracotomy, or by resecting portions of the second and third rib posterior-laterally. A superiorly based rectus muscle flap can be turned over the costal margin and inset to a thoracic defect. Pectoralis major can be delivered into the thoracic cage by removing segments of the second and third rib at approximately the mid-clavicular line (Figs 3 and 4). Omental flaps can either be tunneled over the costal margin to reach mediastinal defects or delivered through the diaphragm to reach thoracic defects.^7^

Pectoralis major is an anterior chest wall muscle attaching the pectoral girdle to the anterior chest wall. It has a clavicular head (arising from the medial aspect of the clavicle) and a sternal head (arising from manubrium, sternum and costal cartilages 1 to 6). These parts converge to form a single wide tendon attaching to the lateral intertubercular groove on the proximal humerus. Its surface markings are the clavicle superiorly, the sternum medially, and the anterior axillary fold laterally. The thoracoacromial trunk is the major pedicle and arises from the second part of the axillary artery. The surface markings of the dominant pedicle is represented by a line drawn from the acromion to the xiphisternum and another drawn vertically down from the junction of the middle and lateral third of the clavicle (Fig 2).^8^ Several minor pedicles also supply the pectoralis major (intercostal perforators from the internal mammary artery and the lateral thoracic artery which anastomosis with the thoracoacromial trunk).

Denervation of pectoralis major requires a thorough understanding of its anatomy. It is innervated by the lateral and medial pectoral nerves so named for their origins from the lateral and medical cords of the brachial plexus. The lateral pectoral nerve runs lateral to the axillary artery and passes inferior to the clavicle with the pectoral branch of the thoracoacromial trunk located medially. It passes through the clavipectoral fascia and lies medial to pectoralis minor. The medial pectoral nerve arises medial to the axillary artery and passes through pectoralis minor via its deep surface. It then sends 2 to 3 branches to pectoralis major. During dissection of the muscle from the deep aspect, the pedicle is protected while the branches of the medial pectoral nerve that traverse pectoralis minor are divided. Branches of the lateral thoracic artery may also need to be divided at this time as they enter the deep surface of the flap. As the lateral pectoral nerve runs with the vascular pedicle, it is dissected and ligated at this time.^7^

The patient described in this case had a pectoralis major muscle flap delivered into the thoracic cavity through a window created by removing a section of the third rib (Figs 3 and 4).
